# The AxBioTick study – immune gene expression signatures in human skin bitten by *Borrelia*-infected versus non-infected ticks

**DOI:** 10.1186/s12879-024-10279-2

**Published:** 2024-12-18

**Authors:** Nellie Carlströmer Berthén, Samuel Cronhjort, Marika Nordberg, Per-Eric Lindgren, Marie Larsson, Peter Wilhelmsson, Johanna Sjöwall

**Affiliations:** 1 The Borrelia Research Group of the Åland Islands, the Åland Islands, Mariehamn, Finland; 2 Division of Inflammation and Infection, Department of Biomedical and Clinical Sciences, Linköping University, Linköping, Sweden; 3 Åland’s Health Care, the Åland Islands, Mariehamn, Finland; 4 Laboratory Medicine, Department of Clinical Microbiology, Region Jönköping County, Jönköping, Sweden; 5 Division of Molecular Medicine and Virology, Department of Biomedical and Clinical Sciences, Linköping University, Linköping, Sweden

**Keywords:** Skin, Human, Tick bite, *Borrelia*, Immune response, Transcriptome

## Abstract

**Background:**

*Borrelia* infection is caused by *Borrelia burgdorferi* sensu lato and transmitted by *Ixodes ricinus* ticks, a common tick-borne infection in Northern Europe. The establishment of *Borrelia* infection depends on transmission of the spirochetes, as well as the immune response generated in the skin after a bite. Here we aim to investigate the local immune response in the skin after a tick bite and assess the possible direct effects of *Borrelia*, by applying gene expression analysis of the immune response in skin exposed to *Borrelia*-infected and non-infected ticks, respectively.

**Methods:**

Skin biopsies from the study participants were taken 7–10 days after the tick-bite. The ticks and skin biopsies were analysed by real-time PCR for *Borrelia* spp. and other tick-borne pathogens. Dermal transcriptome profiles derived from RNA sequencing with focus on immune system regulation were created. In addition, we performed enrichment analysis of dermal transcriptome profiles with focus on immune system regulation.

**Results:**

Skin biopsies exposed to a *Borrelia*-positive tick induced an overall higher expression of immune-related genes. Cytokines involved in the regulation of T-cell and macrophage activation, pro-inflammatory regulators and Toll-like receptor 2, 3 and 7 involved in pathogen recognition were upregulated in skin exposed to *Borrelia*, although *Borrelia* DNA was not detected in the biopsies.

**Conclusion:**

The evidence of upregulation of genes in *Borrelia* exposed skin suggests an influence on the immune system of ticks and spirochetes. Characterization of *Borrelia*-associated gene expression signatures in the skin could contribute to future diagnostics and increase our understanding of the development of various manifestations of *Borrelia* infection.

**Supplementary Information:**

The online version contains supplementary material available at 10.1186/s12879-024-10279-2.

## Background

*Borrelia burgdorferi* sensu lato (*B.b*.s.l.) is the most common tick-borne pathogen in Europe, transmitted by the *Ixodes ricinus* tick [1]. Several steps are involved in the transmission of *Borrelia* bacteria from the tick to the skin. Studies conducted in mouse models have indicated that tick feeding for more than 24 h increases the risk of transmission to the skin [2]. However, the minimum attachment time of the tick required for successful transmission to humans has not yet been determined. Nymphs are the most common developmental stage of ticks to bite humans. However, pathogen content of ticks increases with the developmental stage, causing the highest risk of infection in humans bitten by an adult, engorged tick [3–5].

*Borrelia* infection can manifest in many ways, with *erythema migrans* (EM) as the most common. EM is classified as an early infection with an expanding local erythema in the skin [6]. The immunological processes involved in the pathogenesis of *Borrelia* infection after a tick bite are still not fully understood. A study presented by Glatz et al., revealed that macrophages and dendritic cells are the first and predominant cells found in tick bitten skin, and mRNA levels of CXC chemokine ligand (CXCL) 1, CXCL8, Interleukin (IL) 1-β, IL-5 and Toll-like receptor (TLR) 2 have been shown to increase [7]. However, tick saliva has a counteracting effect on this early immune response, by suppressing the expression of pro-inflammatory factors and stimulating the upregulation of IL-10 receptor, a known mediator of the anti-inflammatory cytokine IL-10 [8]. However, histological examination of EM reveals a perivascular infiltration dominated by T-Lymphocytes followed by CD68 + macrophages [9] and a local expression of IL-10, Interferon (IFN)-γ, Tumor necrosis factor (TNF) a, IL-1β and IL-6 and upregulation of genes encoding CXCL9, CXCL19 and CXCL11 [10]. In fact, an early pro-inflammatory activation in the skin positively impacts the clinical prognosis of persisting symptoms [11]. Comparing the gene expression in skin bitten by a *Borrelia*-positive tick with skin bitten by a *Borrelia*-negative tick could provide valuable insights into the specific immune responses triggered by the *Borrelia* bacteria, leading to a better understanding of the development of infection and *Borrelia* pathogenesis. Additionally, it could potentially aid in the early diagnosis of *Borrelia* infection.

## Materials and methods

### Aim

The aim of the present study was to investigate the local immune response in the skin following a tick bite and assess the potential impact of *Borrelia* on this response. This was achieved by RNA sequencing of immune genes in skin biopsies exposed to ticks infected with *Borrelia* and ticks not infected with *Borrelia*, respectively.

### Study design

The AxBioTick study is a prospective study conducted during 2018–2022 on the Åland Islands with the purpose of characterizing risk factors for tick-borne infections in tick bitten individuals. In short, at inclusion, ticks and blood samples were collected and a questionnaire was filled in regarding the participant’s general health, previous tick-borne diseases, and the anatomical location of the tick bite (S1), which was marked with a pencil. Skin punch biopsies from the tick bite site and an opposite body site were taken 7–10 days after inclusion. After eight weeks, blood samples were drawn, and any additional detached ticks were collected. A second questionnaire regarding health status and symptoms was filled in (S2). A concluding follow-up interview by phone was performed four months after inclusion. The study design has been described elsewhere [12].

### Study participants

In the present study as a part of the AxbioTick-study, we used skin biopsy samples from six participants included in the AxBioTick-study, who had been bitten by a *Borrelia*-positive tick, as confirmed by PCR (ct value 30.29- 37.03) and matched them with six participants of similar age and sex who had been bitten by a *Borrelia*-negative tick. All participants were asymptomatic and without current medication.

### Handling, storage, and analyses of ticks

On the collection day, the ticks were frozen at −75°C. On the day of tick nucleic acid extraction, the developmental stage of each tick was morphologically determined. The blood feeding time was estimated using the method previously described by Gray et al. [13]. The tick species was identified through molecular analysis [14]. Subsequently, the ticks underwent real-time PCR analysis to detect the presence of various pathogens, including *B.b.*s.l, *Borrelia miyamotoi*, Tick-borne encephalitis virus (TBEV), *Rickettsia* spp., *Neoehrlichia mikurensis*, *Anaplasma phagocytophilum* and *Babesia* spp., and species were determined by conventional PCR-assays, as previously described [14].

### *Borrelia* antibody analyses

Serum samples drawn at inclusion and eight weeks after inclusion were analysed in parallel with an in-house enzyme-linked immunosorbent assay (ELISA) to detect *Borrelia* specific C6 peptide IgG1 antibodies (*Borrelia* C6IgG1). Seroconversion was defined as a change from seronegative to seropositive or an increase in arbitrary antibody units (AU) between the two samples of at least 32.3% (sample 1 <2.10 AU) or 18.3% (sample 1 2.10–30 AU) or 9.5% (sample 1 >30 AU) [12, 15].

### Skin biopsies

#### Handling and storage

Under sterile conditions, a 4 mm skin punch biopsy was taken from the tick bite site of each participant and a 2 mm skin biopsy from the opposite side of the body. The biopsies were placed in 500 µL RNA-later for 16–20 h and frozen in −75°C after removal of RNA-later. The skin biopsies selected in this study were divided into two groups, 1) skin bitten by a *Borrelia-*positive tick (*Borrelia* exposed, BEXP) and their individual skin biopsies taken from the opposite side of the body in relation to the tick-bite (controls), 2) skin bitten by a *Borrelia*-negative tick (*Borrelia* unexposed, BUNEXP) and their individual controls.

#### RNA-purification and sequencing

The biopsies were weighed, and the tissue was disrupted and homogenized individually by bead-beating in 2 ml safe-lock microcentrifuge tubes (Eppendorf AG, Hamburg, Germany) with a 5 mm stainless steel bead (Qiagen, Hilden, Germany) in 600 μl RLT buffer (Qiagen) using a TissueLyser II (Qiagen) for 2 min at 25 Hz, and total RNA was purified using Qiagen RNEasy Mini Kit according to manufacturer’s protocol “Purification of Total RNA from Animal Tissues”. Total RNA was quantified after purification using NanoDrop (Thermo Fisher Scientific, Massachusetts, US), and quality and integrity was determined by electrophoresis using Agilent RNA 6000 Nano kit (Agilent, California, US). Purified total RNA was stored at –70°C. Libraries were prepared from total RNA using Illumina TruSeq Stranded mRNA (poly-A selection), and sequencing was performed on NovaSeq 6000 at NGI Sweden.

#### Molecular detection of Borrelia spp. in the skin biopsies

The RNA extracted from the biopsies was subjected to reverse transcription to generate cDNA, utilizing the Illustra™ Ready-to-Go RT-PCR Beads kit (GE Healthcare, Amersham Place, UK) in accordance with the manufacturer’s instructions. Subsequently, the samples underwent real-time PCR analysis to assess transmission of *Borrelia* in the skin biopsies. A previously described genus-specific real time PCR targeting the *Borrelia* spp. 16S rRNA gene was used to detect the pathogen [14].

### Bioinformatics and statistical analyses

RNA reads from Next generation sequencing (NGS) were trimmed, aligned and quantified using the nf-core RNAseq pipeline [16]. A principal component analysis (PCA) was performed on the reads from the NGS. Differential gene expression analysis was performed with DESeq2 [17] independently comparing BEXP and BUNEXP with their controls. Fold-change (FC) shrinkage of the result of the differential gene expression was performed using apeglm [18].

The effect of patient’s gender and *Borrelia* on the results of the differential gene expression was estimated using permutational multivariate analysis of variance using distance matrices [19]. The impact of continuous variables such as the patient’s age, time from tick bite to biopsy and the tick’s estimated blood feeding time on the differential expression was estimated using envfit [19]. The level of significance was set to < 0.05 for all statistical analyses, after correction for multiple testing.

Gene set enrichment analysis was performed, analysing data from the RNA sequencing using QIAGEN Ingenuity Pathway analysis (IPA) software. (QIAGEN Inc., https://www.qiagenbioinformatics. com/products/ingenuity-pathway-analysis) [20]. IPA analysis was generated from the output from the DESeq2 analysis. The two groups (BEXP and BUNEXP) were analyzed separately. Upstream regulators and canonical pathways analysis with focus on immune responses and top regulator analysis were performed. The data set was set to include 4112 *Borrelia*-negative and 4139 *Borrelia*-positive analysis-ready molecules with log FC lower than −0.5 or higher than 0.5. The top twenty upstream regulators and canonical pathways with the greatest difference in z-score were selected for the report.

## Results

### Study participants

Seven out of twelve study participants were females, and the median age was 69 years (range 39–75 years). The initial *Borrelia* IgG-seropositivity among the participants was 75%. Three (25%) participants seroconverted in *Borrelia* C6IgG1 eight weeks after the tick bite. Ten (83%) of the participants reported either to be asymptomatic or did not develop new symptoms eight weeks after the tick bite, one (8.3%) developed EM, one (8.3%) suffered from arthralgia. Among the three participants who seroconverted, two were asymptomatic and one developed arthralgia. Two of them were bitten by a *Borrelia*-negative tick, and their additional ticks were also negative. However, both reported to have had tick-bites earlier the same season (Table 1, S3).

**Table 1:** Characteristics, self-reported symptoms, and diagnosis among the participants

	**Participants bitten by a;**	
	***Borrelia*-positive tick**	***Borrelia*-negative tick**
	*n* = 6	*n* = 6
**Gender**		
Male	3	2
Female	3	4
** Age** median (range)	65 (39–72)	65 (57–75)
** Tick-bitten earlier the same season**	6	6
** Seropositivity at inclusion**	5	4
*** Borrelia* C6IgG1 antibody seroconversion after eight weeks**	1	2
**Symptoms after eight weeks**		
Asymptomatic or no new symptoms	5	5
Arthralgia	0	1
** Symptoms after four months**		
Asymptomatic	4	5
Myalgia/arthralgia	1	1
Nausea	0	1
Loss of appetite	0	1
Shortness of breath	1	0
**Diagnosis under study period**		
* Erythema migrans*	1	0

Four months after the initial tick bite, nine (75%) were asymptomatic. Three participants sought medical care for their symptoms, and one was diagnosed with EM. The participant with arthralgia recovered with unprescribed anti-inflammatory treatment and the participant with EM developed undiagnosed myalgia and arthralgia. Another participant developed myalgia and arthralgia and one developed nausea, loss of appetite and shortness of breath (Table 1, S3).

### Tick characteristics

Six participants were bitten by a *Borrelia* PCR-positive tick, whereof two were *B. afzelii*, two *B. garinii,* one *B. valaisiana,* and one untyped *Borrelia* species. The other six participants were bitten by a *B.b.*s.l. PCR-negative tick. All the ticks were of the specie *I. ricinus and* both nymphs and adult females were represented developmental stages. The estimated median blood feeding time was 24h (range 9 –59 h) (Table 2) (S3). None of the ticks tested positive for any other tick-borne pathogens.

**Table 2:** Characteristics of the detached ticks

	**Participants bitten by a;**	
	***Borrelia*-positive tick**	***Borrelia*-negative tick**
***Borrelia* spp. in ticks**		
* B. afzelii*	2	0
* B. garinii*	2	0
* B. valaisiana*	1	0
Untyped *Borrelia* spp.	1	0
** Estimated blood-feeding time** median h (range)	23 (9–59)	24 (10–34)
**Tick developmental stage**		
Adults /nymphs	2/4	1/5
*** Borrelia* PCR positive skin biopsies**	0	0

### Skin biopsies

The RNA concentration was higher in the tick bitten biopsies (median: 182 ng/uL, range: 59.8–404.5) than in the controls (median: 16.9 ng/uL, range: 6.4–22.1). However, the RNA integrity number (RIN) showed high quality RNA in both the tick bitten biopsies and controls (median: 9.6, range: 6.7–10). One control did not produce a signal and was excluded from the analysis. All the biopsies from individuals bitten by *Borrelia* PCR-positive ticks were negative for *Borrelia* in the real-time PCR.

#### Gene expression profiles in skin biopsies

**Fig. 1:**
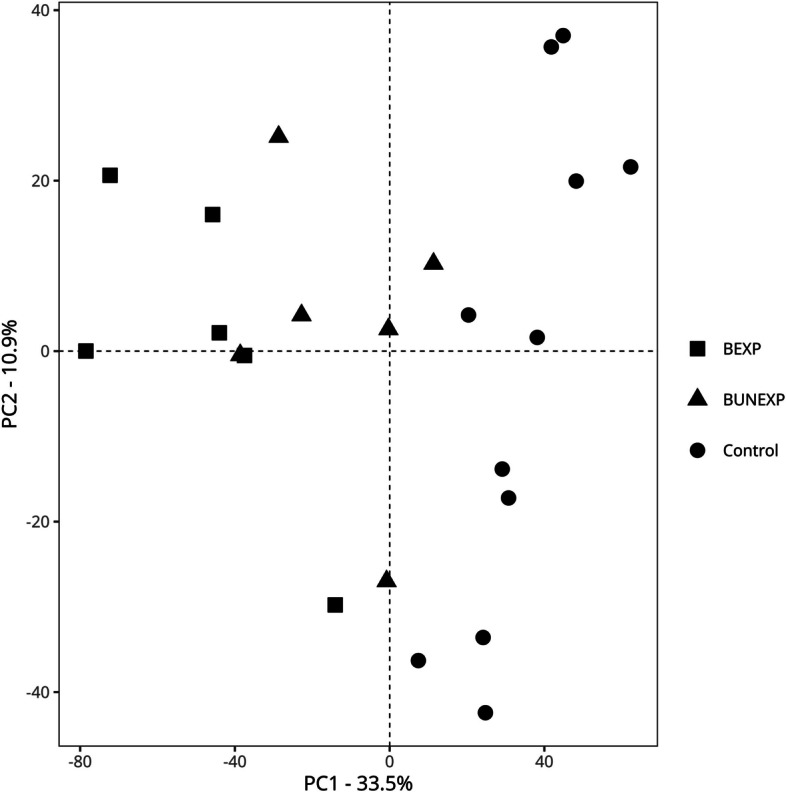
Principal component analysis. A principal component analysis (PCA) performed in DESeq2 (version: 1.34) in R (version 4.1.3) with skin bitten by a *Borrelia*-positive tick (BEXP) as squares, skin bitten by a *Borrelia*-negative tick (BUNEXP) as triangles and unbitten skin controls as circles

Unsupervised PCA (Fig. 1) revealed that the first principal component accounted for 33.5% of the variation in the data set and distinguished tick bitten biopsies from controls with a significance of *P* < 0.001. However, the differences in the gene data set comparing BEXP and BUNEXP were not statistically significant in permutational analysis *P* = 0.07. The patient characteristics (gender, age, time from tick bite to skin biopsy and the estimated blood feeding time of the tick) did not significantly differ between BEXP and BUNEXP.

Differential gene expression analysis comparing BEXP, and their individual controls identified 3439 (10%) genes as significantly differentially expressed with a logarithmic fold change (LFC) > 0, and 2636 (7.8%) with a LFC < 0 (data not shown). Differential gene expression analysis comparing BUNEXP, and their individual controls identified 2592 (7.9%) genes as significantly differentially expressed with a LFC > 0, and 1755 (5.3%) with a LFC < 0 (S4).

#### Transcriptome gene set enrichment analysis

**Fig. 2:**
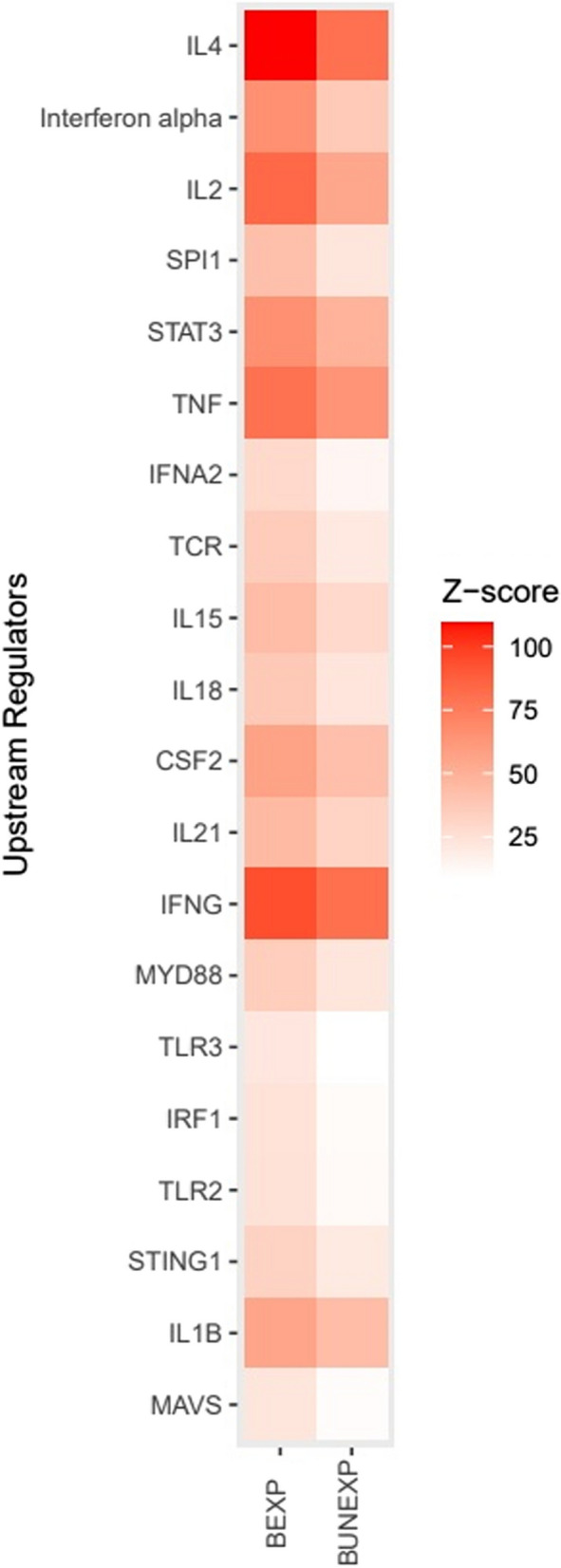
Twenty top upstream regulators. Heatmap with the twenty top upstream regulators from highest to lowest absolute difference in z-scores focusing on immunological responses expressed in skin bitten by a *Borrelia*-positive tick (BEXP) and skin bitten by a *Borrelia*-negative tick (BUNEXP)

**Fig. 3:**
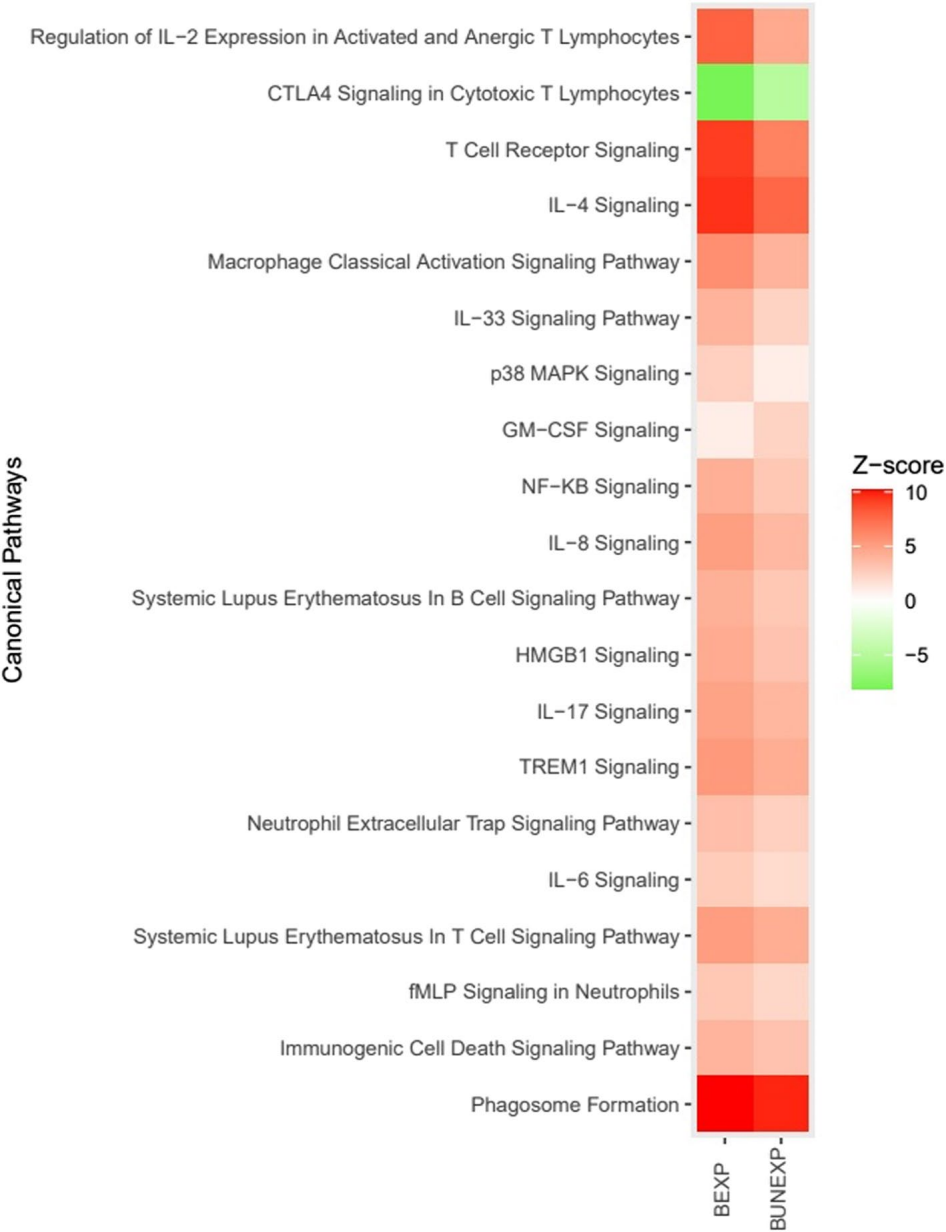
Twenty top canonical pathways. Heatmap with the twenty top canonical pathways from highest to lowest absolute difference in z-scores focusing on immunological responses expressed in skin bitten by a *Borrelia*-positive tick (BEXP) and skin bitten by a *Borrelia*-negative tick (BUNEXP)

Even though there was no significant separation between BEXP and BUNEXP in the principal component analysis, we proceeded with IPA analysis to visualize the genes with significantly different expression levels between BEXP and its controls and BUNEXP and its controls, respectively. Predicted data from the IPA analysis indicated that the BEXP biopsies had more elevated z-scores compared to BUNEXP biopsies, which suggested an overall increased inflammatory response (Figs. 2 and 3). The top twenty upstream regulators in BEXP revealed an elevated activation of type I IFNs and signalling pathways involved in their activation compared to BUNEXP. In addition, compared to BUNEXP, more genes were activated of upstream regulators involved in T cell and NK cell responses such as IL-4, IFN-γ, IL-18 and IL-21 in BEXP skin (Fig. 2). The largest absolute z-score difference in the upstream regulators was observed in the expression of IL-4 which was higher in BEXP skin compared to BUNEXP skin followed by IFN-α, IL-2, Spi-1 proto-oncogene (SPI1), Signal transducer and activator of transcription (STAT) 3 and TNF. Moreover, there were an increased expression of the pathogen recognition receptors TLR2, TLR3 and TLR7 observed in BEXP (Fig. 2, S5) compared to BUNEXP skin. The upstream regulator with the largest relative difference between the two groups was the activation of Immunity-Related GTPase M (IRGM), which was 222.3% more activated in the BEXP group (S5).

All canonical pathways with positive Z-score, besides GM-CSF signalling, were predicted to be more activated in the BEXP group compared to BUNEXP skin (Fig. 3). The Cytotoxic T-lymphocyte associated protein (CTLA) 4 Signalling in Cytotoxic T Lymphocytes was the only of the top 20 canonical pathway predicted to be inhibited in both groups (Fig. 3). The pathways with highest absolute difference in z-score between BEXP and BUNEXP were seen for the Regulation of IL-2 Expression in Activated and Anergic T Lymphocytes, CTLA4 Signalling in Cytotoxic T Lymphocytes, T Cell Receptor Signalling and IL-4 Signalling (Fig. 3). However, the largest relative differences were seen in the pathway p38 MAPK Signalling which was 179.6% more activated in the BEXP group followed by Regulation of IL-2 Expression in Activated and Anergic T Lymphocytes with a 74.2% difference (S5).

**Fig. 4:**
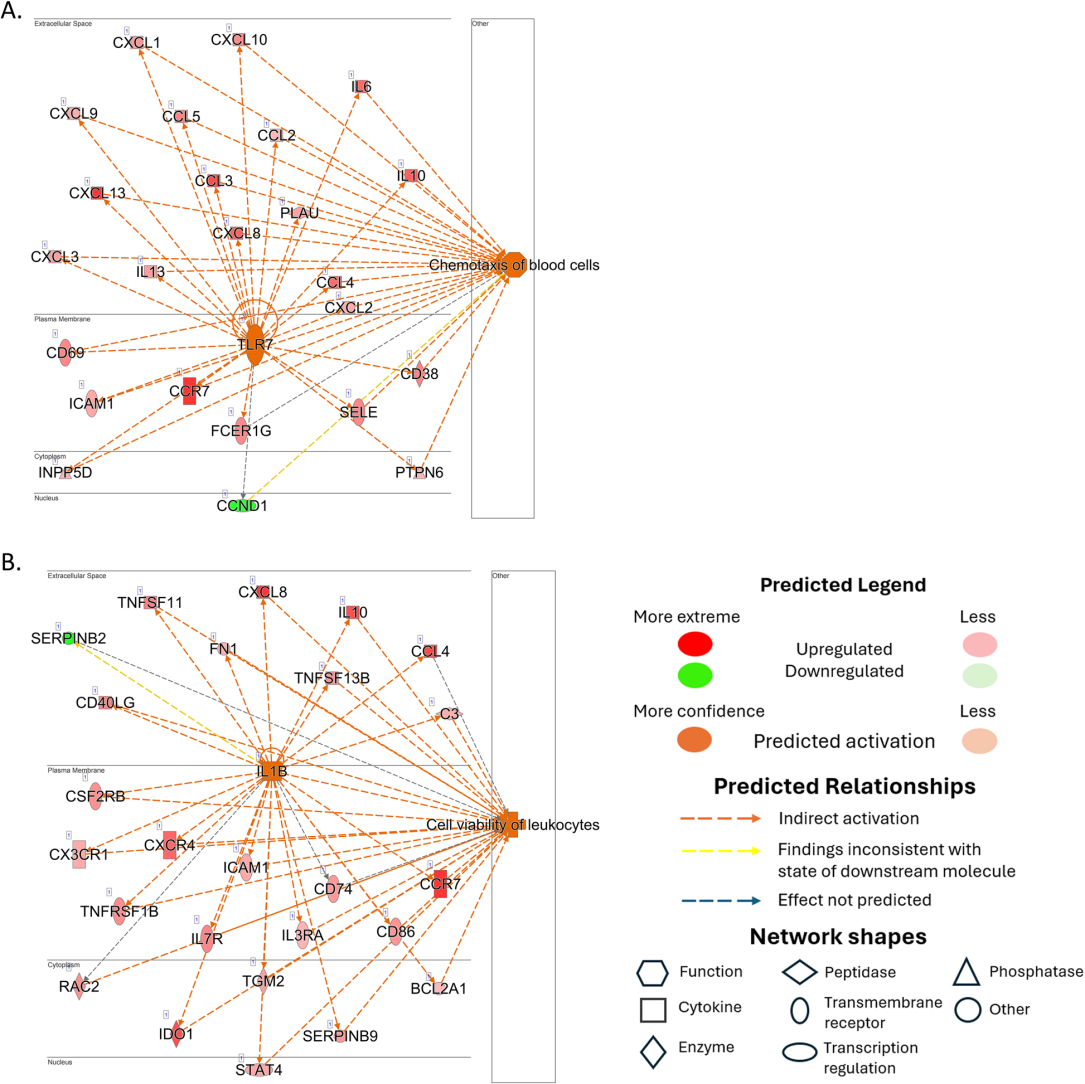
Regulator Network analysis.Ingenuity Pathway analysis (IPA) shows regulator networks displaying top regulators with data set molecules and predicted functions. **A** Predicted top regulator network in skin exposed to a *Borrelia*-positive tick (BEXP) (**B**) Predicted top regulator network in skin exposed to a *Borrelia*-negative tick (BUNEXP)

The results of the top regulator network analysis for BEXP revealed a central role for TLR7, while IL-1β was found to be a significant factor in regulation of genes in BUNEXP (Fig. 4). Specifically, in the BEXP group, the predicted activation of TLR7 was linked to the activation of C–C chemokine receptor (CCR) 7, C–C chemokine ligand (CCL) 3, CXCL13, CXCL8, and IL-6, causing chemotaxis of blood cells. In this group, Cyklin D (CCND) was the only downregulated regulator (Fig. 4). On the other hand, in BUNEXP, the predicted activation of CCR7, CXCL8, IL-10, CCL4, Indoleamine (IDO) 1, and TNF superfamily (TNFSF) 11, induced by IL-1β was expected to lead to cell viability of leukocytes, while SERPINB2 showed a decreased signal in this group.

## Discussion

In this study, no statistically significant differences were found in permutational analysis of gene expression between skin exposed to bites of *Borrelia*-positive and *Borrelia*-negative ticks. However, the IPA suggests that there may be differences worth exploring, which could provide insights into how *Borrelia* affects the host immune response at this early stage. Although the findings are based on a limited number of tissue samples, they contribute to our understanding of the interaction between *Borrelia* and the immune system in the skin.

The innate immune response is the body's first line of defence involving the activation of various cells, such as phagocytes, dendritic cells, mast cells, and NK cells. A crucial aspect of this response is the formation of neutrophil extracellular traps (NETs) in tissues. These NETs trap pathogens, allowing phagocytes to eliminate them. For instance, studies on mouse models have shown that NETs effectively eliminate *Borrelia* bacteria in the skin [21]. Additionally, research has demonstrated that innate immune activation predominates in skin directly after tick detachment [7]. Accordingly, in our study we could confirm activation of innate immune responses in tick-bitten skin by findings of upregulated expression of the NETs pathway, IFNs and TNFs especially in the *Borrelia* exposed group. However, the GM-CSF pathway, which regulates the proliferation of monocytes, dendritic cells, and macrophages, was surprisingly predicted to be less activated in the same group in comparison to the unexposed group. In earlier studies on blood samples, have shown increased activation of GM-CSF in peripheral blood mononuclear cells (PBMC) and serum after exposure to *Borrelia* spirochetes [22, 23]. Our findings suggest that *Borrelia* bacteria might reduce GM-CSF signalling in human skin, potentially decreasing coiling phagocytosis; a technique used by phagocytes to eliminate *Borrelia*, which is induced by GM-CSF [24]. On the other hand, IL-1β, an innate pro-inflammatory cytokine involved in recruiting neutrophils and inducing IL-6 and TNF-α, was predicted to have a vital role in tick bitten skin, regardless of *Borrelia* exposure. This is consistent with findings in a study conducted by Glatz et al., in which IL-1β was found to be significantly increased in skin biopsies analysed directly after tick detachment [7]. Moreover, the p38 MAPK signalling was predicted to be more activated in the *Borrelia* exposed group. This pathway stimulates the activation of proinflammatory regulators such as TNF-α and IL-1β. p38 MAPK has been shown to be activated in mice injected with *Borrelia* spirochetes and that the activation of p38 MAPK is associated with production of proinflammatory cytokines caused by the *Borrelia* bacteria [25]. The highest relative difference in upstream regulator values between the two groups was found for IRGM, which was more upregulated in the *Borrelia* exposed group. IRGM, a GTPase protein, plays a crucial role in activating intracellular immunity by fostering autophagy formation, which eliminates intracellular bacteria. The increased activation of IRGM observed in the *Borrelia* exposed group signifies an increase in intracellular immune activation.

Toll-like receptors are proteins that play a vital role in activating the innate immune system by recognizing molecules originating from microorganisms. Unexpectedly, despite no detected transmission of *Borrelia,* we found increased expression of TLR7, TLR3 and TLR2 in *Borrelia* exposed skin, possibly as indirect evidence of *Borrelia* exposure or presence in the skin. In fact, TLR2 is known to be involved in tick bite recognition [7]. Previous studies analysing PBMCs cultured together with *Borrelia* have indicated that TLR7 plays a significant role in the recognition of *Borrelia* spirochetes [26]. TLR2 and TLR3, although important for recognition of *Borrelia*, may be supressed by tick saliva [7]. It is known that TLR2 and TLR3 upon activation stimulate the activation of pro-inflammatory regulators, such as IL-8 and TNF-α [8, 27]. These regulators were also upregulated in the *Borrelia* exposed group.

Once early clinical manifestations of *Borrelia* infection occur, such as EM, the adaptive immune response plays a dominant role. This involves the infiltration of T-lymphocytes and the upregulation of cytokines such as CXCL9, CXCL10, and CXCL11, which are important for T-cell migration [9, 10, 28]. In our study, skin biopsies taken 7–10 days after a tick bite still showed activation of both innate and adaptive immune responses. Notably, regulators associated with T-cell signalling, such as IL-2, IL-4, IFN-γ and STAT3 were upregulated, particularly in the *Borrelia* exposed group. This indicates a predominant activation of T-cell signalling pathways including downregulation of CTLA4 signalling. The biggest absolute difference in Z-score between *Borrelia* exposed and unexposed skin was observed in the activation of IL-4, which was more pronounced in *Borrelia* exposed skin biopsies. This finding is interesting, since IL-4 is known to have limited effectiveness in eliminating *Borrelia* spirochetes [29]. Despite the absence of *Borrelia* DNA in the skin biopsies, the observed IL-4 gene expression still might have an association with *Borrelia* exposure besides counteracting excess inflammation in the *Borrelia* exposed skin.

We observed a higher presence of pro-inflammatory rather than anti-inflammatory responses in tick bitten skin prior to development of any skin erythema. This is in line with previous studies, which have shown that tick bitten skin, in general, is mainly dominated by pro-inflammatory regulators such as CCL2, CCL3, CCL4, CXCL1, CXCL8, IL-1β and TNF-α [7], whereas skin from EM has been shown to express elevated levels of both pro- and anti-inflammatory responses [9–11, 28]. This could be an effect of the tick saliva, which stimulates pro-inflammatory cytokines in fibroblasts, and the fact that strong pro-inflammatory responses are typically counterbalanced by anti-inflammatory responses over time [8].

According to our analyses, the skin biopsies exposed to *Borrelia*-infected ticks did not exhibit any detectable *Borrelia* DNA. There can be several reasons for this. The risk of transmitting *Borrelia* spirochetes through a tick bite is low, but the risk increases with the duration of the tick's blood-feeding. In our study, the median feeding time was 23 h which is considered an uncertain time for transmission since no minimum tick feeding time is established [3, 30]. A study conducted by Kern et al. demonstrated that in mouse models the peak presence of *Borrelia* establishment in the skin occurs between 5–15 days after a tick bite [31]. This suggests, in our samples, either unsuccessful transmission or successful elimination of the spirochetes by the immune system. Another theory might be that migration of the bacteria has occurred in the skin and therefore are undetected at the bite site 7–10 days after the bite. Nevertheless, Grillon et al. observed that even low numbers of spirochetes, undetectable by PCR, can be transmitted to the skin and trigger local immune responses [32]. This phenomenon could not be ruled out in our skin biopsies. The seropositivity of *Borrelia* C6IgG1 antibodies was 75% in this study group and the group is often exposed to ticks. When individuals are frequently exposed to ticks the immune system has encountered antigens from ticks and tick-borne pathogens multiple times. This repeated exposure may lead to a primed immune response upon subsequent tick bites.

## Conclusion

We conclude that skin exposed to *Borrelia* shows overall higher upregulation of genes associated with both innate and adaptive immune responses 7–10 days after tick-bite, compared to skin exposed only to tick bites. Our study is too small to draw comprehensive conclusions about the impact of *Borrelia* on the immune responses in the skin and to analyse the individual immune differences regarding the clinical outcome, which is why larger studies are necessary to confirm our findings. Nonetheless, the observed differences are noteworthy. Identifying immune regulators associated with *Borrelia* exposure could enhance the understanding of *Borrelia* infections and might improve early diagnosis.

## Supplementary Information

Supplementary Material 1.

Supplementary Material 2.

Supplementary Material 3.

Supplementary Material 4.

Supplementary Material 5.

## Data Availability

The dataset used in the current study are available in the article and from the S3-Supplemantary-file-3. Individual data regarding the participants are available from the corresponding author on reasonable request.

## Abbreviations

B.b.sl.: *Borrelia Burgdorferi* Sensu lato
BEXP: *Borrelia* Exposed skin (Skin bitten by a *Borrelia*-positive tick)
BUNEXP: *Borrelia* Unexposed skin (Skin bitten by a *Borrelia*-negative tick)
CCND: Cyclin D
CCR: C-C Chemokine Receptor
cDNA: Complementary Deoxyribonucleic Acid
CTLA: Cytotoxic T-Lymphocyte Antigen
CXCL: CXC chemokine ligand
C6IgG1: C6 Immunoglobulin G1
DNA: Deoxyribonucleic Acid
ELISA: Enzyme-Linked Immunosorbent Assay
EM: *Erythema* *migrans*
FC: Fold Change
GM-CSF: Granulocyte-Macrophage Colony-Stimulating Factor
GTPase: Guanosine Triphosphatase
IDO: Indoleamine 2,3-Dioxygenase
IFN: Interferon
IL: Interleukin
IPA: Ingenuity Pathway analysis
IRGM: Immunity-Related GTPase Family M
LFC: Log Fold Change
MAPK: Mitogen-Activated Protein Kinase
mRNA: Messenger Ribonucleic Acid
NETs: Neutrophil Extracellular Traps
NGS: Next-Generation Sequencing
NK: Natural Killer
PBMC: Peripheral Blood Mononuclear Cells
PCA: Principal Component Analysis
PCR: Polymerase Chain Reaction
RIN: Ribonucleic Acid Integrity Number
RNA: Ribonucleic Acid
SPI1: Spi-1 proto-oncogene
STAT: Signal Transducer and Activator of Transcription
spp.: Species
TLR: Toll-Like Receptor
TNF: Tumor Necrosis Factor
TNFSF: Tumor Necrosis Factor Superfamily
