# Supplementary material for: The AxBioTick study – immune gene expression signatures in human skin bitten by Borrelia-infected versus non-infected ticks
Source: BMC Infect Dis. 2024 Dec 18;24:1422. doi: 10.1186/s12879-024-10279-2 (PMC11654342; doi:10.1186/s12879-024-10279-2)
Supplement: Supplementary file 1 — Supplementary Material 1. [file 12879_2024_10279_MOESM1_ESM.pdf]

AxBioTick, Inclusion Date_____________ Study code:______________

Do you have any of these diseases?

Asthma: Yes ☐ No ☐ Don't know ☐

Allergy: Yes ☐ No ☐ Don't know ☐

Diabetes: Yes ☐ No ☐ Don't know ☐

Tumor disease: Yes ☐ No ☐ Don't know ☐

If yes, when? _________ What type of tumor and where is it located?

____

Chronic inflammatory disease: Yes ☐ No ☐ Don't know ☐

If you answered yes above, please specify the disease and treatment/medication:

____

Are you being treated with cortisone? Yes ☐ No ☐ Don't know ☐

If yes, what dose and for how long? ____

Are you being treated with biological anti-inflammatory drugs? Yes ☐ No ☐ Don't know ☐

If yes, which drug? ____

Are you taking any other medications? Yes ☐ No ☐ Don't know ☐

If yes, which drug, dose, and treatment duration?_________________________________

____

For which condition(s) are you taking the above medications?

____

____

Vaccinations

Are you vaccinated against TBE (Tick-borne encephalitis)? Yes ☐ No ☐ Don't know ☐

If yes, year-month-day: _______ ____ ____ Number of doses: ___________

When was the last dose given? Year-month-day: _______ ____ ____

Are you vaccinated against Yellow Fever? Yes ☐ No ☐ Don't know ☐

If yes, year-month-day: _______ ____ ____

Are you vaccinated against Japanese Encephalitis? Yes ☐ No ☐ Don't know ☐

If yes, year-month-day: _______ ___ ___

Tick- information

When did you notice that you had been bitten by a tick?

Year-month-day: _______ ___ ___ Around what time: _______

When do you think you were bitten by a tick?

Year-month-day: _______ ___ ___ Around what time: _______

In which location were you when you were bitten by a tick?__________________________

____

Where were you staying at that time?

Near lake/sea ☐ In the forest ☐ In the garden ☐ On the lawn ☐

Other, namely: ____

When was the tick removed?

Year-month-day: _______ ___ ___ Time: _______

Did you remove the entire tick? Yes ☐ No ☐ Don't know ☐

Have you been bitten by a tick earlier this season? Yes ☐ No ☐ Don't know ☐

If yes, how many times?

1-4 ☐ 5-9 ☐ 10 or more ☐

AxBioTick, Inclusion Date_____________ Study code:______________

Where on the body was the tick located?

____

____

Specify also on the body map below.

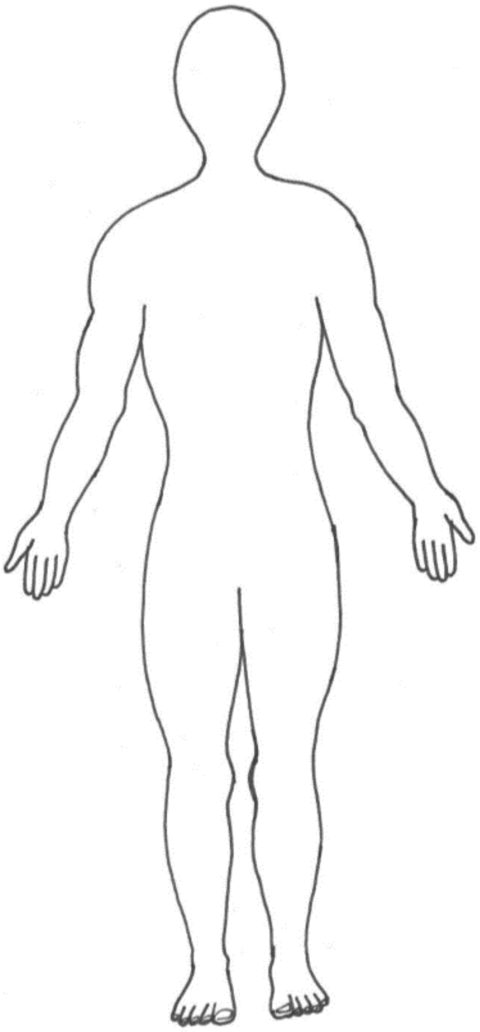

Front ☐ Back ☐

Have you previously been treated for a tick-borne infection (e.g., Lyme disease)?

Erythema migrans (> 5 cm in diameter): Yes ☐ No ☐ Don't know ☐

If yes, how many times? ___ When? _________________

Did you receive antibiotics for this? Yes ☐ No ☐ Don't know ☐

If yes, which one? _________________________________

AxBioTick, Inclusion   Date_____________ Study code:______________

Arthritis/Lyme arthritis: Yes ☐ No ☐ Don't know ☐

If yes, how many times? ___ When? _________________

Did you receive medication for this? Yes ☐ No ☐ Don't know ☐

If yes, which one? _________________________________

Neuroborreliosis (Lyme infection of the nervous system): Yes ☐ No ☐ Don't know ☐

If yes, how many times? ___ When? _________________

Did you receive medication for this? Yes ☐ No ☐ Don't know ☐

If yes, which one? _________________________________

Unspecific/disseminated borreliosis: Yes ☐ No ☐ Don't know ☐

If yes, how many times? ___ When? _________________

Did you receive medication for this? Yes ☐ No ☐ Don't know ☐

If yes, which one? _________________________________

Notes: ____

____

Have you previously been treated for a tick-borne infection like Ehrlichiosis/Anaplasmosis/Tick fever?

Yes ☐ No ☐ Don't know ☐

If yes, how many times? ___ When? _________________

Did you receive medication for this? Yes ☐ No ☐ Don't know ☐

If yes, which one? _________________________________

Have you previously been treated for a tick-borne infection like TBE?

Yes ☐ No ☐ Don't know ☐

If yes, how many times? ___ When? _________________

AxBioTick, Inclusion   Date_____________ Study code:______________

Do you smoke? Yes ☐ No ☐ Quit ☐ year _____

If yes, how many cigarettes per week? _____________

How many years have you smoked? _______________________

Do you have any pets? Yes ☐ No ☐

Dog: Yes ☐ No ☐

Cat: Yes ☐ No ☐

Rabbit: Yes ☐ No ☐

Other animal, specify: ____

Interview conducted by: ____
