# Supplementary material for: The AxBioTick study – immune gene expression signatures in human skin bitten by Borrelia-infected versus non-infected ticks
Source: BMC Infect Dis. 2024 Dec 18;24:1422. doi: 10.1186/s12879-024-10279-2 (PMC11654342; doi:10.1186/s12879-024-10279-2)
Supplement: Supplementary file 2 — Supplementary Material 2. [file 12879_2024_10279_MOESM2_ESM.pdf]

AxBioTick, Eight-week follow-up Date:__________ Study number:_____________

Have you received new tick bites since the previous sampling?

Yes ☐ No ☐ Don't know ☐

If yes, when? Year-month-day: ______________

Are these ticks collected in the study? Yes ☐ No ☐

How have you been feeling since the previous sampling?

Have you felt as usual? Yes ☐ No ☐ Don't know ☐

If no, indicate if you have experienced any of the following symptoms:

Newly appeared rash/skin change: Yes ☐ No ☐ Don't know ☐

Where? ____

Erythema migrans: Yes ☐ No ☐ Don't know ☐

Where? ____

Headache: Yes ☐ No ☐ Don't know ☐

Fatigue: Yes ☐ No ☐ Don't know ☐

Fever 38°C or higher: Yes ☐ No ☐ Don't know ☐

Chills: Yes ☐ No ☐ Don't know ☐

Neck pain: Yes ☐ No ☐ Don't know ☐

Poor appetite: Yes ☐ No ☐ Don't know ☐

Nausea/vomiting: Yes ☐ No ☐ Don't know ☐

Weight loss: Yes ☐ No ☐ Don't know ☐

Dizziness: Yes ☐ No ☐ Don't know ☐

Difficulty concentrating: Yes ☐ No ☐ Don't know ☐

Radiating pain – severe nighttime pain: Yes ☐ No ☐ Don't know ☐

Where? ____

Muscle or joint pain: Yes ☐ No ☐ Don't know ☐

Where? ____

Numbness/tingling: Yes ☐ No ☐ Don't know ☐

AxBioTick, Eight-week follow-up Date:__________ Study number:_____________

Where? ____

Facial paralysis (half/whole face): Yes ☐ No ☐ Don't know ☐

Other? ____

If symptoms were indicated above, how many days did they last?

If symptoms were indicated above, did you seek medical care for the symptom?

Yes ☐ No ☐

If yes, which clinic did you visit? ______________________________________

Date of the visit Year-month-day: __________________

If you described any symptoms above, did they occur before or after any new tick bite?

Before ☐ After ☐ Don't know ☐

During the study period, have you started or completed vaccination for TBE?

Yes ☐ No ☐

If yes, when? Year-month-day: ________ ____ ____

Which dose? ______

During the study period, have you started or completed any other vaccination?

Yes ☐ No ☐

If yes, what did you vaccinate against? ________________________

During the study period, have you taken antibiotics or started new medication?

Yes ☐ No ☐

If yes, when? Year-month-day: ______ ____ ____

Which medication? _____________

Interview conducted by: _______________________________________
