# Supplementary material for: The AxBioTick study – immune gene expression signatures in human skin bitten by Borrelia-infected versus non-infected ticks
Source: BMC Infect Dis. 2024 Dec 18;24:1422. doi: 10.1186/s12879-024-10279-2 (PMC11654342; doi:10.1186/s12879-024-10279-2)
Supplement: Supplementary file 3 — Supplementary Material 3. [file 12879_2024_10279_MOESM3_ESM.pdf]

Supplementary Table S4. A summary of tick and study participant characteristics., self-reported symptoms, and diagnosis eight-weeks (+/- 2 weeks) and four months after tick-bite.

	**Participants**							**Ticks**								
								**Inclusion tick**				**Additional ticks[2]**			**Tick bites reported**	
	Sex	Age	Diagnosis	Reported symptoms 8 weeks	Reported symptoms 4 months	Seropositive at inclusion	Seroconversion anti-*Borrelia* C6 IgG1 after 8 weeks	Stage	*Borrelia* PCR	*Borrelia* spp.	Feeding duration[1]	No. of collected additional ticks	*Borrelia* PCR positive	*Borrelia* spp.	Before inclusion	Within study
**BUNEXP**	F	69	-	-	-	+	-	N	-		34	4	1/4	B.g	+	+
	M	75	-	-	-	+	-	A	-		10	0			+	+
	F	61	-	-	-	+	+	N	-		18	3	0/3		+	+
	M	73	-	a	-	+	+	N	-		22	2	0/2		+	+
	F	67	-	-	-	-	-	N	-		26	0			+	+
	F	57	-	-	a,b,c,d	-	-	N	-		34	1	0/1		+	+
**BEXP**	F	72	-	-	e	+	-	N	+	B. v	30	8	2/8	B.a, B.g	+	+
	M	69	-	-	-	+	+	N	+	B. a	59	0			+	+
	M	72	-	-	-	-	-	A	+	B. g	11	0			+	-
	F	39	-	-	-	+	-	A	+	UT	9	1	0/1		+	+
	F	65	EM	f	a,b	+	-	N	+	B. a	24	0			+	+
	M	70	-	-	-	+	-	N	+	B. g	45	19	3/19	UT/UT/UT	+	+

Abbreviations: F; female, M; male, EM; *erythema migrans*, +; positive. -; negative, N; nymph, A; adult female tick, B. a.; *Borrelia afzelii*; B. g.: *Borrelia garinii*; B. v.: *Borrelia valaisiana*, UT; untypeable

a) arthralgia, b) myalgia, c) loss of appetite, d) nausea, e) shortness of breath, f) skin lesion
Blank space; not analyzed

1. Estimated tick feeding time [13]

2. Ticks detached from the participants during the study period
