# Supplementary material for: The AxBioTick study – immune gene expression signatures in human skin bitten by Borrelia-infected versus non-infected ticks
Source: BMC Infect Dis. 2024 Dec 18;24:1422. doi: 10.1186/s12879-024-10279-2 (PMC11654342; doi:10.1186/s12879-024-10279-2)
Supplement: Supplementary file 5 — Supplementary Material 5. [file 12879_2024_10279_MOESM5_ESM.pdf]

Supplementary file 1: Z-scores from the Ingenuity Pathway analysis (IPA)

Canonical Pathways	Borrelia negative	Borrelia positive	Absolute difference	Difference %
p38 MAPK Signaling	0,905	2,53	1,625	179,558
Regulation of IL-2 Expression in Activated and Anergic T Lymphocytes	4,491	7,825	3,334	74,237
IL-33 Signaling Pathway	2,294	3,962	1,668	72,711
CTLA4 Signaling in Cytotoxic T Lymphocytes	-4,808	-8,1	-3,292	68,469
NF-κB Signaling	2,858	4,2	1,342	46,956
T Cell Receptor Signaling	6,351	9,146	2,795	44,009
Macrophage Classical Activation Signaling Pathway	4,012	5,774	1,762	43,918
IL-6 Signaling	1,886	2,668	0,782	41,463
Systemic Lupus Erythematosus In B Cell Signaling Pathway	2,887	4,064	1,177	40,769
fMLP Signaling in Neutrophils	2,111	2,887	0,776	36,760
Neutrophil Extracellular Trap Signaling Pathway	2,528	3,441	0,913	36,116
HMGB1 Signaling	3,207	4,359	1,152	35,921
IL-8 Signaling	3,71	5,014	1,304	35,148
MIF-mediated Glucocorticoid Regulation	2	2,646	0,646	32,300
T Cell Exhaustion Signaling Pathway	1,789	2,335	0,546	30,520
IL-17 Signaling	3,78	4,849	1,069	28,280
Complement System	1,667	2,111	0,444	26,635
IL-4 Signaling	7,55	9,43	1,88	24,901
TREM1 Signaling	4,264	5,292	1,028	24,109
Immunogenic Cell Death Signaling Pathway	3,162	3,873	0,711	22,486
NUR77 Signaling in T Lymphocytes	2,646	3,162	0,516	19,501
MIF Regulation of Innate Immunity	2,236	2,646	0,41	18,336
FAT10 Cancer Signaling Pathway	2,236	2,646	0,41	18,336
Systemic Lupus Erythematosus In T Cell Signaling Pathway	4,315	5,096	0,781	18,100
Leukocyte Extravasation Signaling	3,9	4,564	0,664	17,026
Th2 Pathway	4,841	5,547	0,706	14,584
Cytotoxic T Lymphocyte-mediated Apoptosis of Target Cells	2,646	3	0,354	13,379
Neuroinflammation Signaling Pathway	5,284	5,988	0,704	13,323
Fcγ Receptor-mediated Phagocytosis in Macrophages and Monocytes	3,873	4,359	0,486	12,548
iNOS Signaling	2	2,236	0,236	11,800
Role of Pattern Recognition Receptors in Recognition of Bacteria & Viruses	3,638	4,025	0,387	10,638
Role of NFAT in Regulation of the Immune Response	5,831	6,403	0,572	9,810
Dendritic Cell Maturation	5,741	6,26	0,519	9,040
Natural Killer Cell Signaling	3,888	4,221	0,333	8,565
Crosstalk between Dendritic Cells and Natural Killer Cells	4,899	5,303	0,404	8,247

Production of Nitric Oxide and Reactive Oxygen Species in Macrophages	4,472	4,796	0,324	7,245
NF-κB Activation by Viruses	2,828	3	0,172	6,082
Th1 Pathway	6,091	6,429	0,338	5,549
Macrophage Alternative Activation Signaling Pathway	5,98	6,301	0,321	5,368
Phagosome Formation	9,695	10,107	0,412	4,250
IL-15 Production	3,606	3,71	0,104	2,884
CD28 Signaling in T Helper Cells	3,771	3,838	0,067	1,777
Fc Epsilon RI Signaling	3,464	3,5	0,036	1,039
PKCθ Signaling in T Lymphocytes	5,292	5,303	0,011	0,208
Calcium-induced T Lymphocyte Apoptosis	4,123	4,123	0	0,000
CD40 Signaling	2,333	2,333	0	0,000
Inflammasome pathway	2,236	2,236	0	0,000
PD-1, PD-L1 cancer immunotherapy pathway	-3,545	-3,528	0,017	-0,480
S100 Family Signaling Pathway	8,232	8,159	-0,073	-0,887
ICOS-ICOSL Signaling in T Helper Cells	4,899	4,849	-0,05	-1,021
IL-2 Signaling	2,646	2,53	-0,116	-4,384
IL-7 Signaling Pathway	2,53	2,309	-0,221	-8,735
Th17 Activation Pathway	2,111	1,897	-0,214	-10,137
GM-CSF Signaling	2,333	0,905	-1,428	-61,209
Interferon Signaling	N/A	2,121		
Toll-like Receptor Signaling	0	2,121	2,121	

**Upstream Regulators**	**Borrelia negative**	**Borrelia positive**	**Absolute difference**	**Difference %**
IRGM	3,800	12,247	8,447	222,300
TREX1	5,354	15,888	10,534	196,763
RNASEH2B	3,767	10,911	7,144	189,647
IFNL1	5,276	14,580	9,304	176,361
RIPK2	4,472	11,715	7,243	161,964
TICAM1	6,947	16,806	9,859	141,927
IFN alpha/beta	7,798	18,167	10,369	132,958
TLR3	9,657	22,047	12,390	128,309
SLC15A4	6,943	15,725	8,782	126,492
PRKD1	4,908	10,596	5,688	115,894
TRAF3IP2	6,304	13,141	6,836	108,435
IFNA2	14,415	29,381	14,967	103,831
TSLP	5,785	11,773	5,988	103,495
MAVS	11,152	22,667	11,515	103,258

IRF1	12,025	24,096	12,070	100,378
PDCD1	7,001	13,902	6,900	98,560
USP18	7,640	14,755	7,115	93,120
TLR2	12,899	24,888	11,989	92,945
APP	7,227	13,937	6,711	92,864
IRF7	9,083	17,419	8,336	91,775
EBI3	6,879	12,898	6,019	87,490
SPI1	22,545	42,238	19,693	87,347
NONO	9,945	18,262	8,317	83,627
IFNA1/IFNA13	5,940	10,781	4,841	81,491
NFATC2	12,160	21,960	9,800	80,597
Interferon alpha	36,987	65,811	28,824	77,931
HMGB1	7,485	13,276	5,791	77,368
IFIH1	5,788	10,063	4,275	73,867
Ifnar	9,278	15,895	6,617	71,315
Ifn	10,619	18,152	7,533	70,938
TCR	21,134	36,042	14,908	70,542
cytokine	16,640	27,848	11,208	67,357
MOG	8,099	13,498	5,400	66,671
NfkB1-RelA	6,989	11,630	4,641	66,413
IL7	16,487	27,131	10,644	64,564
TLR7	14,479	23,506	9,027	62,348
CHROMR	7,182	11,633	4,451	61,980
IL18	23,187	37,518	14,331	61,808
TLR4	16,852	27,120	10,268	60,931
IFNA4	7,666	12,242	4,577	59,703
RIGI	13,494	21,317	7,824	57,979
PARP1	8,621	13,610	4,989	57,869
RELA	17,753	27,778	10,025	56,468
IKBKB	13,367	20,893	7,526	56,300
MYD88	22,623	35,279	12,656	55,942
STING1	20,831	32,451	11,620	55,785
PSMB11	10,539	16,286	5,747	54,533
IKZF2	8,681	13,397	4,716	54,329
IL3	17,880	27,542	9,663	54,043
IL2	54,694	83,404	28,710	52,491
VEGFA	11,547	17,561	6,015	52,088

OLR1		6,872	10,428	3,556	51,739
RNASE1		7,285	11,005	3,720	51,060
IL1RN		10,569	15,965	5,396	51,057
ZBTB10		12,260	18,471	6,211	50,665
CTLA4		9,058	13,646	4,588	50,652
IL15		29,401	44,269	14,868	50,572
IFNB1		12,587	18,902	6,315	50,175
TERT		9,502	14,186	4,685	49,303
CSF3		18,458	27,536	9,078	49,184
Nfat (family)		9,798	14,598	4,800	48,990
PPP2CA		7,866	11,659	3,793	48,216
IFNAR1		12,866	19,049	6,183	48,057
CHUK		10,619	15,684	5,065	47,699
GATA2		16,851	24,882	8,031	47,659
OSM		11,282	16,636	5,353	47,451
IFN Beta		17,359	25,516	8,157	46,993
TCF3		14,999	21,951	6,953	46,356
SOCS1		11,265	16,362	5,096	45,239
CCL11		9,590	13,909	4,319	45,036
RNASE2		7,639	11,063	3,424	44,824
IL5		15,299	22,116	6,817	44,561
TLR9		15,321	21,940	6,619	43,201
Tlr		8,038	11,448	3,410	42,431
SMARCA5		7,306	10,360	3,054	41,807
IL21		32,001	45,301	13,300	41,560
S100A9		9,927	14,044	4,117	41,475
Klrk1		8,853	12,480	3,627	40,967
ETS1		15,368	21,568	6,200	40,348
BTK		13,390	18,747	5,357	40,009
IRF3		7,844	10,967	3,123	39,812
NFATC1		8,677	12,087	3,410	39,297
Ap1		9,610	13,302	3,692	38,422
Ige		20,597	28,499	7,902	38,364
IL12 (family)		19,814	27,365	7,551	38,112
SMAD3		11,695	16,079	4,385	37,494
Jnk		11,547	15,838	4,291	37,166
CD36		8,198	11,233	3,035	37,026

FN1	8,034	11,005	2,971	36,984
TRADD	7,944	10,867	2,923	36,802
BCR (complex)	13,473	18,427	4,954	36,768
IL10RA	15,182	20,723	5,542	36,505
C5	10,806	14,727	3,921	36,288
IL4	80,041	108,892	28,851	36,045
ITK	10,724	14,580	3,856	35,954
STAT3	48,657	66,088	17,431	35,825
CD2	8,755	11,853	3,099	35,392
IL1	21,512	28,948	7,436	34,568
NFKB1	21,278	28,286	7,008	32,937
CSF2	42,875	56,832	13,957	32,553
BHLHE40	18,458	24,452	5,994	32,474
NfkB-RelA	8,125	10,685	2,560	31,503
ITPR2	15,303	20,087	4,784	31,260
CSF1	34,090	44,604	10,514	30,840
IL-1R	11,565	14,970	3,405	29,443
TNFSF13B	20,843	26,977	6,134	29,430
IL27	24,602	31,742	7,139	29,019
CD28	16,487	21,044	4,557	27,643
IGF1	10,424	13,277	2,853	27,369
IL12 (complex)	34,614	44,083	9,470	27,359
FLT3LG	16,471	20,902	4,432	26,906
S100A8	11,946	15,125	3,179	26,610
RRAS2	10,918	13,821	2,903	26,588
CD14	8,651	10,943	2,292	26,501
NFkB (complex)	30,480	38,533	8,053	26,421
IL1B	44,292	55,850	11,558	26,095
TYROBP	18,663	23,227	4,564	24,452
TNF	64,067	79,606	15,539	24,254
BRD4	11,039	13,681	2,642	23,929
IL6	41,956	51,925	9,969	23,761
JUN	13,182	16,286	3,104	23,546
Vegf	8,967	11,051	2,084	23,241
ERK1/2	13,262	16,263	3,001	22,627
TGFB1	38,756	47,403	8,647	22,310
SP1	13,093	15,891	2,798	21,372

Tnf (family)	26,242	31,581	5,339	20,343
CD3	28,567	34,345	5,779	20,228
CITED2	17,610	21,099	3,488	19,808
KLF6	16,266	19,316	3,050	18,751
NFKBIA	14,961	17,747	2,785	18,616
IRF2BP2	17,212	20,270	3,057	17,761
IL6R	11,262	13,250	1,988	17,656
IL32	11,549	13,568	2,020	17,488
TNFSF11	16,301	19,138	2,837	17,406
CXCL12	10,788	12,659	1,871	17,342
TNFSF12	12,935	15,175	2,239	17,313
P38 MAPK	13,877	16,238	2,362	17,019
IFNG	80,581	93,589	13,009	16,144
IL13	48,219	55,850	7,631	15,826
SMARCA4	23,088	26,737	3,649	15,803
Alpha catenin	22,488	25,900	3,411	15,170
CD40	17,837	20,486	2,649	14,853
IL33	47,618	54,145	6,527	13,707
Brd4	15,300	17,391	2,092	13,672
STAT1	34,896	39,401	4,505	12,909
PRDM1	32,612	36,822	4,209	12,907
TGM2	24,202	27,247	3,046	12,585
IL17A	23,753	26,652	2,899	12,204
CD44	10,823	12,102	1,279	11,818
Hbb-b2	16,214	18,115	1,901	11,725
JAG2	10,538	11,698	1,161	11,014
FKBP10	11,125	12,332	1,207	10,847
CG	10,951	12,120	1,169	10,674
CASP4	9,793	10,790	0,997	10,179
PTGS2	17,590	19,366	1,776	10,099
EP300	12,327	13,563	1,236	10,027
Hbb-b1	10,951	11,995	1,044	9,529
IL1A	20,756	22,683	1,926	9,281
PI3K (complex)	11,059	12,070	1,010	9,136
MRGPRX3	10,542	11,417	0,874	8,292
MAPK14	12,822	13,785	0,963	7,512
CEBPB	17,471	18,461	0,990	5,664

F2		12,692	13,329	0,637	5,018
CCL5		11,626	12,201	0,574	4,940
C3		10,060	10,551	0,491	4,885
CD40LG		39,742	41,455	1,714	4,312
Srgn		14,271	14,865	0,595	4,167
LDL		19,793	20,411	0,618	3,124
IKZF1		16,893	17,232	0,339	2,007
ERBB2		10,542	10,689	0,147	1,390
GRN		23,715	23,907	0,193	0,812
CCR2		25,946	25,406	-0,540	-2,082
LEP		10,679	10,220	-0,459	-4,301
EGF		11,695	11,147	-0,547	-4,678
SMPD1		12,944	12,269	-0,675	-5,215
CNTF		11,840	11,147	-0,693	-5,852
NR3C1		17,963	16,414	-1,550	-8,626
NFAT5		12,337	11,193	-1,143	-9,269
ERK		11,781	10,650	-1,130	-9,595
CCL20		13,386	12,085	-1,301	-9,720
AGT		32,176	28,443	-3,734	-11,604
SNCA		11,266	9,909	-1,357	-12,044
HIF1A		11,445	9,975	-1,470	-12,847
CSF		17,687	15,382	-2,305	-13,034
APOE		18,218	15,683	-2,535	-13,916
AHR		36,996	31,548	-5,447	-14,724
MAFB		16,778	14,191	-2,587	-15,419
ELOVL3		11,696	9,870	-1,826	-15,611
CEBPA		19,433	16,328	-3,104	-15,974
TNFRSF1A		12,838	10,781	-2,056	-16,019
MAP3K14		14,573	11,995	-2,578	-17,692
SELPLG		13,171	10,790	-2,381	-18,078
HRG		14,912	11,435	-3,477	-23,318
NFKB2		10,091	7,501	-2,589	-25,661
INSIG1		10,868	7,880	-2,989	-27,499
Nr1h		13,093	9,247	-3,846	-29,374
